# Increased FNDC5 is associated with insulin resistance in high fat‐fed mice

**DOI:** 10.14814/phy2.13319

**Published:** 2017-07-04

**Authors:** Brianne L. Guilford, Jake C. Parson, Caleb W. Grote, Stephanie N. Vick, Janelle M. Ryals, Douglas E. Wright

**Affiliations:** ^1^ Department of Applied Health Southern Illinois University Edwardsville Edwardsville Illinois; ^2^ Department of Anatomy and Cell Biology University of Kansas Medical Center Kansas City Kansas; ^3^ Department of Pharmacy Practice Southern Illinois University Edwardsville Edwardsville Illinois

**Keywords:** Diabetes, HOMA‐IR, irisin, obesity, PGC‐1*α*, UCP1

## Abstract

FNDC5/irisin, has recently been identified as a novel protein that stimulates the “browning” of white adipose by inducing thermogenesis via increased uncoupling protein 1 (UCP1). We tested the hypothesis that high fat diet‐induced prediabetic mice would exhibit increased FNDC5 and this effect would be attenuated by chronic exercise. C57BL/6 mice were randomized into three groups for the 4 week intervention: Standard diet (Std, *n *=* *12), High fat diet (HF,* n *=* *14), or High fat diet and free access to a running wheel (HFEX,* n *=* *14). Body weight, glucose, insulin, and the homeostatic model assessment of insulin resistance (HOMA‐IR) were greater in HF compared to Std and HFEX after the 4 week intervention. In support of our hypothesis, FNDC5 was higher in HF in both skeletal muscle and adipose compared to Std and was lower in adipose only in HFEX compared to HF mice. Following the same pattern, PGC‐1*α* was significantly higher in HF compared to Std in skeletal muscle and significantly lower in HFEX compared to HF in adipose. UCP1 was significantly lower in HFEX versus Std (in skeletal muscle) and versus HF (in adipose). HOMA‐IR was significantly correlated with FNDC5 protein levels in adipose. Increased FNDC5 in adipose and skeletal muscle may be a compensatory mechanism to offset high fat diet‐induced weight gain and insulin resistance by increasing energy expenditure.

## Introduction

Irisin has recently been identified as a novel exercise‐inducible myokine that may hold therapeutic potential for both obesity and diabetes. Fibronectin type III domain‐containing protein 5 (FNDC5) is released from muscle and then cleaved to irisin, the soluble peptide hormone. FNDC5 is upregulated in response to acute exercise (Bostrom et al. 2012; Brenmoehl et al. 2014; Norheim et al. 2014; Nygaard et al. 2015) and chronic exercise (Bang et al. 2014; Norheim et al. 2014) in humans and animals. Exercise‐induced expression of FNDC5/irisin in muscle has been shown to be dependent on increased PGC‐1*α* (Bostrom et al. 2012; Brenmoehl et al. 2014; Norheim et al. 2014).

Irisin's actions include “browning” of white adipose tissue (WAT) via increased uncoupling protein 1 (UCP1) levels, a specialized mitochondrial protein that converts WAT to brown adipose tissue (BAT) by dissipating energy in the form of heat (Bostrom et al. 2012; Wu et al. 2014). Browning of WAT has the potential to increase total energy expenditure (Wu et al. 2014) and suggests a role for FNDC5/irisin in treatment of obesity and obesity‐related diseases such as type II diabetes.

The impact of exercise on FNDC5/irisin is somewhat contradictory and the different views may be due to variability in exercise mode, intensity, frequency, duration, and whether exercise was chronic or acute. Following acute endurance exercise, FNDC5/irisin levels are significantly elevated, resulting in increased energy expenditure in mice (Bostrom et al. 2012; Brenmoehl et al. 2014; Norheim et al. 2014) and humans (Norheim et al. 2014; Nygaard et al. 2015). This increased energy expenditure is proposed to be mediated by increased browning of WAT via increased UCP1 levels (Bostrom et al. 2012; Wu et al. 2014). However, other studies have shown no effect of acute (Czarkowska‐Paczek et al. 2014) or chronic endurance exercise on skeletal muscle FNDC5 or circulating irisin (Hecksteden et al. 2013; Kurdiova et al. 2014; Seo et al. 2014). Norheim et al. demonstrated that combined chronic endurance and resistance training increased both PGC‐1*α* and FNDC5 skeletal muscle mRNA, whereas circulating irisin increased only in response to acute combined endurance and resistance exercise (Norheim et al. 2014). Further study is needed to differentiate the effects of acute and chronic exercise on FNDC5 and irisin.

In addition to its role as an exercise‐inducible myokine, it has been purported that irisin may play a compensatory role as an adipokine released in response to obesity and/or insulin resistance. Mice fed high fat diets have higher serum irisin levels than their lean counterparts (Seo et al. 2014; Wu et al. 2014). Similarly, humans that consume a caloric excess diet and have elevated fasting glucose levels exhibit elevated serum irisin levels (Huerta et al. 2015; Schlogl et al. 2015). Furthermore, plasma irisin levels are positively associated with body mass index (Stengel et al. 2013) as well as fat mass (Pardo et al. 2014). Congruently, weight loss induced by bariatric surgery or diet/exercise intervention has resulted in associated reductions in plasma irisin levels (Huh et al. 2012; Sharma et al. 2012; Crujeiras et al. 2014).

Although initial investigations focused on the impact of exercise on FNDC5/irisin, irisin has recently been proposed to have a compensatory role as an adipokine released in response to metabolic disturbance (Novelle et al. 2013). FNDC5 and irisin have been assessed in the context of a high fat diet (Seo et al. 2014; Wu et al. 2014; Yang et al. 2015), obesity (Stengel et al. 2013; Vamvini et al. 2013), and insulin resistance (Choi et al. 2013; Moreno‐Navarrete et al. 2013; Park et al. 2013; Crujeiras et al. 2014; Ebert et al. 2014; Kurdiova et al. 2014; Sesti et al. 2014; Yang et al. 2015; Hu et al. 2016; Huh et al. 2016; Shi et al. 2016), but results are contradictory. Although several studies have shown irisin to be positively associated with insulin resistance (Park et al. 2013; Crujeiras et al. 2014; Ebert et al. 2014; Sesti et al. 2014; Huh et al. 2016), others have shown decreased irisin in type 2 diabetic patients (Choi et al. 2013; Moreno‐Navarrete et al. 2013; Hu et al. 2016) and rodents (Yang et al. 2015). It is plausible that these conflicting results are associated with differences in the severity or duration of insulin resistance.

In addition, the majority of previous studies showing an association between FNDC5/irisin and insulin resistance have used an estimate of insulin sensitivity such as an oral glucose tolerance test (Moreno‐Navarrete et al. 2013) or HOMA‐IR (Park et al. 2013; Crujeiras et al. 2014; Ebert et al. 2014; Hu et al. 2016). Only one study has directly assessed insulin's action using insulin signaling via Akt activation (Yang et al. 2015). In light of irisin's potential role as a pharmacotherapeutic treatment for insulin resistance, it is critical to understand irisin's role in insulin resistance. Here, we tested the hypothesis that high fat diet‐induced prediabetic mice increases FNDC5 and this effect would be attenuated by chronic exercise. Importantly, we report the effects of a high fat diet and exercise on skeletal muscle Akt activation and FNDC5 protein expression in adipose and skeletal muscle.

## Methods

### Animals, diet, and voluntary exercise

Male 7‐week old C57BL/6NCrl mice were obtained from Charles River (Washington, MA) and were kept in controlled illumination on a 12‐h light: 12‐h dark cycle at the University of Kansas Medical Center laboratory animal resources facility. Mice were randomized to one of three treatment groups that are abbreviated throughout as follows: standard diet sedentary (Std), high fat diet sedentary (HF), and high fat diet exercise (HFEX). Sedentary mice were housed two per cage in a pathogen‐free vivarium and had ad libitum access to both water and food that included either a standard diet (8604; Harlan Teklad, Madison Wisconsin; 14% kcals from fat, 32% from protein, and 54% from carbohydrates) or a high fat diet (07011; Harlan Teklad; 54% kcals from fat comprised of both vegetable shortening and corn oil, 21% protein, and 24% kcals from carbohydrate).

Exercised mice were caged individually and had ad libitum access to a running wheel for the entire 4 weeks of the study. The Vital View Data Acquisition System (Mini Mitter, Bend, OR) was used to record each wheel revolution in thirty‐minute intervals and quantify daily running distance. Daily running distance was calculated by multiplying the number of wheel turns by the diameter of the wheel in a 24 h period. Mice were removed from running wheels 24 h prior to tissue harvest in order to eliminate the acute effects of exercise.

All procedures were approved by the University of Kansas Medical Center Animal Use and Care Committee and were compliant with the National Research Council of the National Academies' Guide for the Care and Use of Laboratory Animals (2011).

### Glucose, insulin, and HOMA‐IR

Following a 3‐h fast, blood was collected at baseline and at the end of the 4‐week study immediately before insulin stimulation. Samples were assayed for glucose using glucose diagnostic reagents (Sigma, St. Louis, MO). For insulin measurements, blood was collected into a heparinized microcapillary tube, then pipetted into an Eppendorf tube, and centrifuged for 15 min at 3000***g***. Plasma was removed and frozen at −80°C. Insulin levels were measured using ELISA (mouse insulin Elisa, Alpco, Salem, NH). Fasting insulin and fasting glucose levels were used to calculate the homeostatic model assessment of insulin resistance (HOMA‐IR), a measure of insulin resistance. The following equation was used to calculate HOMA‐IR values: Blood (Glucose (mg/dL) × (Serum Insulin (μU/mL)/405 (Kim et al. 2008).

### Insulin stimulation

Following a three hour fast, sterile PBS (vehicle) or 10 U/kg Humulin R insulin was administered to mice from the SS, HS, and HE groups. Humulin R insulin was diluted to 0.001 U/*μ*L in sterile PBS and 10 *μ*L/g was injected via a single intra‐peritoneal injection. Thirty minutes after insulin injection, mice were anesthetized with isofluorane and decapitated. Blood was collected from the decapitation pool for post‐insulin stimulation glucose quantification.

### Western immunoblotting

After the 30‐min vehicle PBS or insulin stimulation period, mice were euthanized as described above and the gastrocnemius muscle and epididymal fat pad were snap frozen in liquid nitrogen, and stored at −80°C. Only tissues from PBS injected mice were used for the FNDC5, UCP1, and PGC‐1*α* western blots. Tissues were lysed with cell lysis buffer (137 mmol/L NaCl, 20 mmol/L Tris [pH 8.0], 1% NP40, and 10% glycerol) containing protease inhibitors (0.5 mm sodium vanadate, 1 *μ*g/mL leupeptin, 10 *μ*g/mL aprotinin, and 1 mmol/L PMSF) and protein was extracted while tissue lysates were kept on ice for 1 h and vortexed every 10 min. Samples were then centrifuged at 10,000***g*** for 10 min at 4^°^C and the protein concentration of the supernatant was measured with a Bradford assay (Bio‐Rad, Hercules, CA). Samples were prepared to achieve equivalent protein concentrations then boiled at 95–100°C with Lane Marker Reducing Sample Buffer (Thermo Scientific, Waltham, MA) for 3 min. Samples were separated by electrophoresis (35 mA/gel, 0.75 h, 4°C) on 4–15% gradient tris‐glycine polyacrylamide gels (Bio‐Rad). Samples for Akt quantification were transferred onto nitrocellulose membranes (400 mA, 1.5 h, 4°C) and samples for FNDC5, UCP1, and PGC‐1*α* were transferred onto polyvinylidene difluoride (PVDF) membranes using Bio‐Rad Trans Blot Turbo (2 gels/cassette, 25 mA, 7 min). Membranes were blocked for 1 h at room temperature in blocking solution (5% non‐fat powdered milk and 0.05% Tween‐20 in 0.1 mol/L phosphate buffered saline [PBST, pH 7.4]), followed by overnight incubation (4°C) in primary antibody diluted in blocking solution (1% non‐fat powdered milk and 0.05% 0.1 mol/L PBS (pH 7.4)**.**

Membranes were probed using following antibodies: total Akt (C67E7 rabbit mAb #4691; Cell Signaling Technology, Danvers, MA), phospho (Ser473) Akt (rabbit mAb #4060; Cell Signaling), FNDC5 (ab174833; Abcam, Cambridge, MA), PGC‐1*α* (ab72230, Abcam), UCP1 (ab10983; Abcam), *α*‐tubulin (T‐5168), and *β*‐actin (#5125; Cell Signaling). Following PBST washes, membranes were incubated at room temperature for 1 h at room temperature using goat anti‐rabbit horseradish peroxidase‐conjugated (HRP) secondary antibodies (sc‐2004; Santa‐Cruz Biotechnology, Dallas, TX). All bands were visualized by enhanced chemiluminescence (ECL) using Supersignal West (Femto or Pico) Substrate (Pierce, Rockford, IL). Total Akt and pAkt bands were detected on X‐ray film, and quantified via densitometry using NIH Image J software. FNDC5, UCP1, and PGC‐1*α* bands were detected and quantified using Bio‐Rad ChemiDoc MP Imaging System with Image Software Lab.

### Statistical analysis

Data are presented as a mean ± SEM. Data were analyzed using a two‐factor analysis of variance (ANOVA) or repeated measures ANOVA with Fisher's test of least square difference post hoc comparisons. Pearson correlations using two‐tailed *P*‐values were performed to assess the relationship between FNDC5 protein levels in skeletal muscle and adipose and body weight, body weight change, fasting blood glucose, and HOMA‐IR. Statistical significance was set at *P *<* *0.05.

## Results

### Bodyweight, glucose, insulin

**Figure 1:**
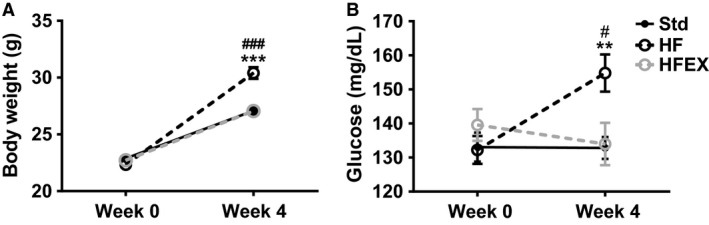
Exercise prevented excess weight gain and prevented elevated glucose levels in high fat‐fed mice. (A) Body weight and (B) Blood glucose. Data are presented as mean ± SEM (*n *=* *19–21 mice per group). ^#^ *P* < 0.05 and ^###^ *P* < 0.001 for Std versus HF; **P < 0.01 and ***P < 0.001 for HF versus HFEX.

**Figure 2:**
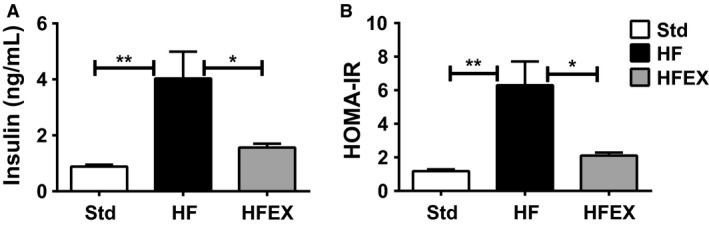
Exercise prevented increased serum insulin and HOMA‐IR in high fat‐fed mice. (A) Serum Insulin and (B) Homeostatic model assessment of insulin resistance (HOMA‐IR). *P < 0.05 and **P < 0.01. Data are presented as mean ± SEM (*n *=* *18–20 mice per group).

Sedentary mice fed the HF diet had increased body weight (Fig. 1A), glucose (Fig. 1B) and insulin levels (Fig. 2A), and HOMA‐IR (Fig. 2B) and greater daily caloric intake compared to Std sedentary mice. Elevations in glucose and insulin levels in HF mice indicated that these mice were prediabetic but not overtly diabetic (Fig. 2B). Body weight (Fig. 1A), glucose (Fig. 1B), insulin (Fig. 2A), and HOMA‐IR (Fig. 2B) in HFEX mice were not different from Std sedentary mice, thus voluntary running wheel exercise prevented deleterious changes in all of these metabolic variables, despite significantly greater daily caloric intake in HFEX (18.4 ± 1.9 kcal/d) compared to Std (12.7 ± 2.5 kcal/d, *P* < 0.001) and HF (14.0 ± 1.1 kcal/d, *P* < 0.001).

### Exercise distance

HFEX mice had ad libitum access to a running wheel in their individual cages 24 h per day, 7 days per week. However, due to their nocturnal nature, mice used the running wheels primarily during the dark portion of the 12 h light/dark cycle from 6 pm to 6 am. On average, all mice ran 9.9 km per 24 h.

### Akt activation

**Figure 3:**
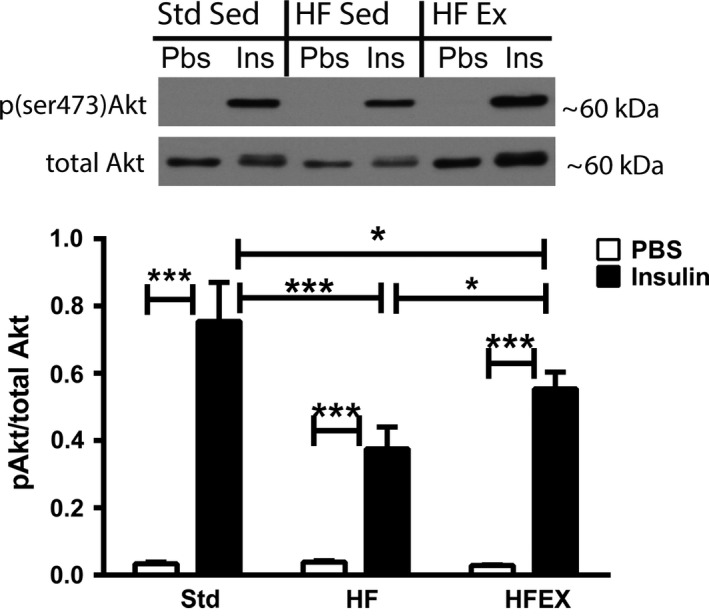
Exercise improves blunted Akt activation in skeletal muscle of high fat‐fed mice. Representative images and quantification of group means for insulin‐stimulated Akt activation in the gastrocnemius. Phosphorylated (ser473) Akt band intensities were normalized to total Akt. Data are presented as mean ± SEM (*n *=* *9–10 mice per group). *P < 0.05, **P < 0.01, and ***P < 0.001.

After insulin stimulation, insulin action was evaluated by quantification of Akt activation (pAkt/total Akt) in the gastrocnemius muscle. In Std, HF, and HFEX, insulin injection elicited a significant increase in Akt activation compared to PBS injection in the corresponding group (Fig. 3). Akt activation was significantly blunted in the gastrocnemius of HF compared to Std mice (Fig. 3). Akt activation was significantly higher in HFEX compared to HF mice, indicating that exercise attenuated the deleterious effects of the high fat diet on insulin signaling in muscle (Fig. 3). However, HFEX mice nevertheless had significantly lower Akt activation than Std, revealing that 4 weeks of exercise did not completely restore Akt activation to normal levels (Fig. 3).

### FNDC5, UCP1, and PGC‐1*α* protein levels

**Figure 4:**
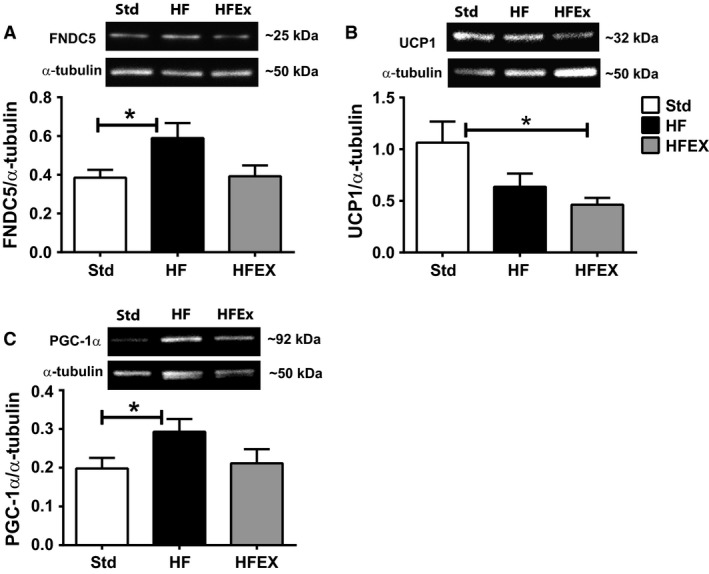
FNDC5, UCP1, and PGC‐1*α* protein levels in the gastrocnemius muscle. Representative Western blot images and quantification of group means for (A) FNDC5, (B) UCP1, and (C) PGC‐1*α*. Band intensities were normalized to *α*‐tubulin. Data are presented as mean ± SEM (*n *=* *7–12 mice per group). *P < 0.05.

In the gastrocnemius muscle, FNDC5 and PGC‐1*α* protein levels were significantly higher in HF compared to the Std mice (Fig. 4A and C). UCP1 levels were higher in the Std compared to HFEX mice (Fig. 4B).

**Figure 5:**
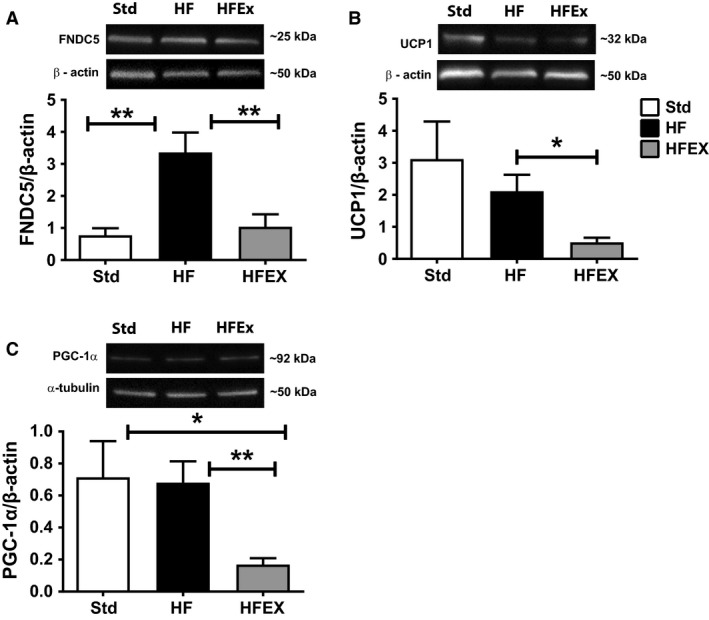
FNDC5, UCP1, and PGC‐1*α* protein levels in epididymal fat. Representative Western blot images and quantification of group means for (A) FNDC5, (B) UCP1, and (C) PGC‐1*α*. Band intensities were normalized to *β*‐actin. Data are presented as mean ± SEM (*n *=* *8–12 mice per group 9–12). *P < 0.05 and **P < 0.01.

Consistent with skeletal muscle, FNDC5 in adipose was significantly higher in HF compared to Std mice (Fig. 5A). In addition, adipose levels of FNDC5, UCP1, and PGC‐1*α* were significantly higher in HF compared to HFEX mice (Fig. 5A–C). Adipose PGC‐1*α* was also significantly higher in Std compared to HFEX mice (Fig. 5C).

### Correlations between FNDC5 and variables associated with insulin resistance

**Figure 6:**
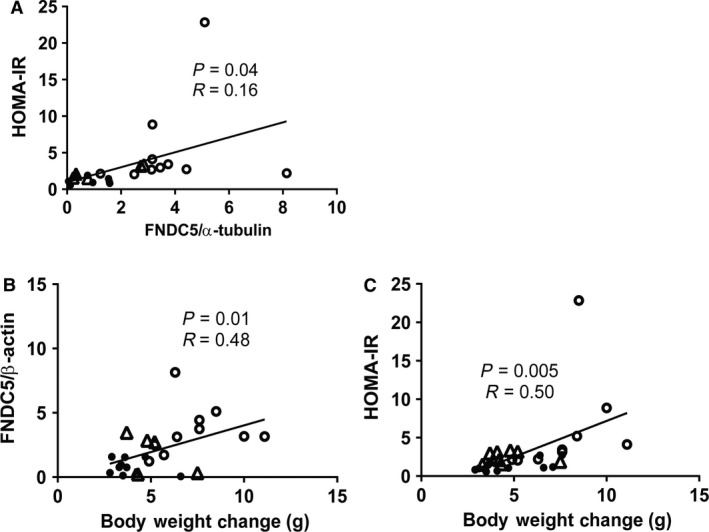
FNDC5 protein levels in adipose are significantly correlated with HOMA‐IR and body weight change. A Pearson correlation using two‐tailed P‐values detected significant positive correlations between adipose FNDC5 protein levels and HOMA‐IR (A) and body weight change (B). In addition, body weight change was correlated with HOMA‐IR (C). Mice from all three diet groups are included in each correlation (*n *=* *23 mice). Solid circles represent Std, open circles represent HF, and open triangles represent HFEX. Note: FNDC5 was not quantified in tissues from the insulin‐injected mice.

Considering the role that activation of the PGC‐1*α*‐FNDC5/irisin pathway may have in response to insulin resistance, Pearson correlations were performed between FNDC5 protein levels in muscle or adipose and body weight, fasting blood glucose, and HOMA‐IR. Neither muscle nor adipose FNDC5 protein levels were significantly correlated with body weight or fasting blood glucose. However, adipose FNDC5 was significantly correlated (*P* = 0.04, *R* = 0.16) with HOMA‐IR (Fig. 6A). In addition, body weight change, an indicator of energy balance, was significantly correlated with adipose FNDC5 (Fig. 6B, *P* = 0.01, *R* = 0.48) and HOMA‐IR (Fig. 6C, *P* = 0.005, *R* = 0.50).

## Discussion

The current experiments reveal the impact of exercise on the FNDC5/PGC‐1*α* pathway in high fat‐fed prediabetic mice with impaired insulin action. FNDC5 was increased in both skeletal muscle and adipose in high fat‐fed, overweight, prediabetic mice compared to mice fed a standard diet. Exercise prevented the increase in body weight, glucose, HOMA‐IR, FNDC5, and attenuated the loss of skeletal muscle insulin action (Akt activation) in high fat‐fed mice. These results indicate a potential compensatory increase in FNDC5 to counteract body weight gain and loss of insulin sensitivity in high fat‐fed mice. Furthermore, our results contribute new evidence that irisin acts in a homeostatic fashion in metabolic conditions such as obesity and prediabetes. However, the precise role of the PGC‐1*α*‐FNDC5/irisin pathway in regulation of energy metabolism remains elusive.

### Exercise did not increase PGC‐1*α*, FNDC5, or UCP1

Irisin was initially identified as a PGC‐1*α*‐dependent exercise‐inducible myokine that stimulated the browning of adipose tissue via increased thermogenesis (Bostrom et al. 2012). Previous studies demonstrate increased FNDC5/irisin in response to chronic exercise (Bostrom et al. 2012; Roca‐Rivada et al. 2013; Norheim et al. 2014; Wu et al. 2014) and increased PGC‐1*α* following acute (Bostrom et al. 2012; Norheim et al. 2014) and chronic (Norheim et al. 2014; Wu et al. 2014) endurance exercise. Conversely, in the current study, 4 weeks of chronic running wheel exercise did not induce a significant increase in FNDC5 or PGC‐1*α* protein levels in skeletal muscle or adipose compared to sedentary mice. PGC‐1*α* in adipose was actually lower in HF fed exercised mice compared to both sedentary mice fed the standard diet or HF diet and this pattern was consistent with lower FNDC5 levels in adipose in the HFEX group compared to HF.

Importantly, others have reported that PGC‐1*α* and FNDC5 peak a few hours after an acute endurance exercise and return to baseline levels within 24 h (Bostrom et al. 2012; Brenmoehl et al. 2014; Norheim et al. 2014; Nygaard et al. 2015). In order to ameliorate the acute effects of exercise, mice in the current study were removed from exercise wheels 24 h before sacrifice. This may, in part, explain the lack of increase in FNDC5 in the exercised mice. In addition, exercised mice in the current study were also on a high fat diet, and previous studies showing increased FNDC5 in exercised mice were in mice fed a standard diet (Bostrom et al. 2012; Brenmoehl et al. 2014). Consistent with our findings, Wu et al. demonstrated endurance training had no effect on skeletal muscle FNDC5 levels in rats fed a high or low fat diet (Wu et al. 2014). Furthermore, Perhaps acute exercise increases PGC‐1*α* and subsequently increases FNDC5 in skeletal muscle. It may be that these effects are transient as PGC‐1*α* and FNDC5 levels return to baseline within hours after exercise, thus explaining the lack of increased skeletal muscle PGC‐1*α* and FNDC5 in the current study.

Notably, skeletal muscle and adipose UCP1 levels were significantly lower in HFEX compared Std mice in muscle and HF mice in adipose. This pattern reflects the lack of increase in muscle PGC‐1*α* and FNDC5 in HFEX (vs. Std) and significantly lower adipose PGC‐1*α* in HFEX (vs. Std and HF) and adipose FNDC5 in HFEX (vs. Std and HF). However, previous studies report exercise training induced increased UCP1 levels predominantly in inguinal white adipose tissue (iWAT), opposed to epididymal adipose, which was used in the current study (Roca‐Rivada et al. 2013; Wu et al. 2014; Claycombe et al. 2015). Interestingly, Roca‐Rivada et al. reported UCP1 in BAT decreased after 3 weeks of endurance exercise (Roca‐Rivada et al. 2013). Furthermore, Wu et al. (2014) reported chronic exercise in rats increased UCP1 content and subsequent browning primarily in subcutaneous adipose tissue, however, these effects were attenuated by a high fat diet.

A progressive reduction in UCP1 in BAT during chronic endurance exercise suggests an adaptive response to chronic exercise. Myo‐adipokine cross‐talk may exist in which exercise initially stimulates browning of adipose tissue, but chronic exercise training eventually results in an adaptive halt with reduction in UCP1 expression due to less “necessity” for energy expenditure. This view agrees with our findings in which HFEX exhibited substantially lower UCP1 levels in adipose and skeletal muscle compared to the Std and HF groups respectively. Thus, exercise may prevent an increase in UCP1 and BAT development, contrary to previous thought. This view is consistent with the idea that the PGC‐1*α*‐FNDC5/irisin pathway may be increased transiently in response to exercise.

### FNDC5 and PGC‐1*α* were increased in high fat‐fed sedentary mice

Although initially identified as a myokine (Bostrom et al. 2012), FNDC5/irisin has also been identified as an adipokine, with increased release stimulated by atypical metabolic disturbances such as obesity and insulin resistance (Kleiner et al. 2012; Roca‐Rivada et al. 2013; Stengel et al. 2013; Crujeiras et al. 2014, 2015; Keuper et al. 2014; Pardo et al. 2014; Wu et al. 2014; Yang et al. 2015; Srinivasa et al. 2016; Xin et al. 2016). FNDC5 is significantly elevated in both subcutaneous and visceral adipose tissue in both genetic‐induced obese and diet‐induced obese rats compared to their lean counterparts (Roca‐Rivada et al. 2013). Subcutaneous adipose secreted and contained higher amounts of FNDC5 highlighting a “beneficial, protective” role of subcutaneous adipose, opposed to visceral adipose which is often associated with obesity and metabolic dysfunction (Roca‐Rivada et al. 2013). In the current study, FNDC5 (in skeletal muscle and adipose) and PGC‐1*α* (in adipose) were increased in HF compared to Std. These results suggest a compensatory increase in FNDC5 in effort to increase energy expenditure to offset increased bodyweight and associated insulin resistance in prediabetic high fat‐fed mice. Consistent with this hypothesis, circulating irisin levels are elevated in obese humans and positively correlate with serum insulin (Stengel et al. 2013). These findings are supported by previous studies indicating that irisin levels are increased during obesity in both rodents and humans (Stengel et al. 2013; Lopez‐Legarrea et al. 2014; Wu et al. 2014; Schlogl et al. 2015; Srinivasa et al. 2016). In particular, Stengel et al. reported a positive correlation between body weight and circulating irisin in obese humans (Stengel et al. 2013). Our results also support Wu et al. who demonstrated that high fat‐fed, sedentary mice have 3.45‐fold higher PGC‐1*α* levels in skeletal muscle than sedentary, low‐fat fed mice (Wu et al. 2014). Taken together, this evidence points to a potential compensatory role of FNDC5/irisin as a myo‐adipokine, opposing excess body weight and the development of insulin resistance in the stages of prediabetes.

### FNDC5 levels were correlated with insulin resistance

Here, we report increased FNDC5 and PGC‐1*α* protein in skeletal muscle and adipose in HF sedentary mice that exhibit increased bodyweight, fasting glucose, insulin, HOMA‐IR and cellular insulin resistance as measured by decreased Akt activation. Importantly, HOMA‐IR was positively correlated with adipose FNDC5 protein levels. To our knowledge, no studies have investigated the effects of both a high fat diet and exercise on FNDC5 that include direct quantification of cellular insulin resistance. Importantly, it should be noted that the significant correlation between HOMA‐IR and adipose FNDC5 is limited by the fact that HOMA‐IR is a surrogate marker of insulin resistance rather than a direct measure such as a hyperinsulinemic‐euglycemic clamp.

Our results are congruent with previous reports in both animals and humans that suggest the PGC‐1*α*‐FNDC5/irisin pathway is upregulated in response to chronically elevated blood glucose as well as HOMA‐IR (Huh et al. 2012; Kleiner et al. 2012; Crujeiras et al. 2014; Keuper et al. 2014; Yang et al. 2015; Xin et al. 2016; Zhang et al. 2016). Additionally, Zhang et al. reported a positive correlation between circulating irisin and HbA1c levels in diabetic humans (Zhang et al. 2016). Furthermore, Crujeiras et al. found that individuals who regain weight after initial weight loss had increased irisin levels corresponding with increased bodyweight, fat mass, fasting glucose, insulin, and HOMA‐IR compared to those maintaining a normal weight (Crujeiras et al. 2014).

Our results indicate that HF feeding resulted in reduced Akt activation and increased PGC‐1*α* and FNDC5 in skeletal muscle and adipose, which were all attenuated by exercise. We postulate that insulin resistance stimulates activation of the PGC‐1a/FNDC5 pathway as a compensatory response in effort to increase insulin sensitivity and attenuate impaired insulin signaling. In support of this idea, Xin et al. reported that HF‐diet induced diabetic mice treated with irisin showed dramatically improved glucose tolerance and glucose uptake with increased translocation of GLUT4 in skeletal muscle along with a myriad of other benefits including increased UCP1 in adipose and increased fatty acid oxidation (Xin et al. 2016). Intriguingly, Yang et al. found that irisin treatment recovered impaired Akt and Erk phosphorylation in palmitic‐acid induced insulin resistant myocytes (Yang et al. 2015). These studies and our results support our hypothesis that FNDC5 levels in both skeletal muscle and adipose tissue are increased as an adaptive response in prediabetic mice to counteract the deleterious effects of excess adiposity and perturb metabolic derangements such as insulin resistance. The present findings suggest that the role of irisin as a protective myo‐adipokine in atypical metabolic situations needs further examination, but provides promising support for the potential pharmacotherapeutic use of irisin in the treatment of obesity and type 2 diabetes.

## Conclusion

A high fat diet and chronic exercise significantly impacts the irisin/PGC‐1*α* pathway, insulin sensitivity, and insulin action in prediabetic mice. FNDC5 and PGC‐1*α* protein levels were elevated in skeletal muscle and adipose in HF sedentary mice and compared to Std sedentary mice (in muscle) and HF exercised mice (in adipose). Importantly, adipose FNDC5 was positively correlated with HOMA‐IR. Taken together, these data indicate that exercise may transiently increase these proteins, however, metabolic disturbances such as high fat diet‐induced obesity and insulin resistance may have a greater role in upregulating this pathway in skeletal muscle and adipose. We propose that PGC‐1*α*‐FNDC5/irisin pathway may be upregulated as a compensatory mechanism to induce browning of adipose and subsequently increase energy expenditure to oppose increased adiposity and insulin resistance. This concept suggests FNDC5/irisin may have an important role as a protective myo‐adipokine opposing the development of insulin resistance in the stages of prediabetes and provides further support for the use of FNDC5/irisin as a potential therapeutic treatment for obesity and related metabolic disorders.

## Conflict of Interest

None declared.

## References

[phy213319-bib-0001] Bang, H. S. , D. Y. Seo , Y. M. Chung , K. M. Oh , J. J. Park , F. Arturo , et al. 2014 Ursolic Acid‐induced elevation of serum irisin augments muscle strength during resistance training in men. Korean J. Physiol. Pharmacol. 18:441–446. 25352765 10.4196/kjpp.2014.18.5.441 PMC4211129

[phy213319-bib-0002] Bostrom, P. , J. Wu , M. P. Jedrychowski , A. Korde , L. Ye , J. C. Lo , et al. 2012 A PGC1‐alpha‐dependent myokine that drives brown‐fat‐like development of white fat and thermogenesis. Nature 481:463–468. 22237023 10.1038/nature10777 PMC3522098

[phy213319-bib-0003] Brenmoehl, J. , E. Albrecht , K. Komolka , L. Schering , M. Langhammer , A. Hoeflich , et al. 2014 Irisin is elevated in skeletal muscle and serum of mice immediately after acute exercise. Int. J. Biol. Sci. 10:338–349. 24644429 10.7150/ijbs.7972 PMC3957089

[phy213319-bib-0004] Choi, Y. K. , M. K. Kim , K. H. Bae , H. A. Seo , J. Y. Jeong , W. K. Lee , et al. 2013 Serum irisin levels in new‐onset type 2 diabetes. Diabetes Res. Clin. Pract. 100:96–101. 23369227 10.1016/j.diabres.2013.01.007

[phy213319-bib-0005] Claycombe, K. J. , E. E. Vomhof‐DeKrey , J. N. Roemmich , T. Rhen , and O. Ghribi . 2015 Maternal low‐protein diet causes body weight loss in male, neonate Sprague‐Dawley rats involving UCP‐1‐mediated thermogenesis. J. Nutr. Biochem. 26:729–735. 25858881 10.1016/j.jnutbio.2015.01.008

[phy213319-bib-0006] Crujeiras, A. B. , M. A. Zulet , P. Lopez‐Legarrea , R. de la Iglesia , M. Pardo , M. C. Carreira , et al. 2014 Association between circulating irisin levels and the promotion of insulin resistance during the weight maintenance period after a dietary weight‐lowering program in obese patients. Metabolism 63:520–531. 24439241 10.1016/j.metabol.2013.12.007

[phy213319-bib-0007] Crujeiras, A. B. , M. Pardo , and F. F. Casanueva . 2015 Irisin: ‘fat’ or artefact. Clin. Endocrinol. (Oxf) 82:467–474. 25287317 10.1111/cen.12627

[phy213319-bib-0008] Czarkowska‐Paczek, B. , M. Zendzian‐Piotrowska , K. Gala , M. Sobol , and L. Paczek . 2014 One session of exercise or endurance training does not influence serum levels of irisin in rats. J. Physiol. Pharmacol. 65:449–454. 24930518

[phy213319-bib-0009] Ebert, T. , D. Focke , D. Petroff , U. Wurst , J. Richter , A. Bachmann , et al. 2014 Serum levels of the myokine irisin in relation to metabolic and renal function. Eur. J. Endocrinol. 170:501–506. 24399249 10.1530/EJE-13-1053

[phy213319-bib-0010] Hecksteden, A. , M. Wegmann , A. Steffen , J. Kraushaar , A. Morsch , S. Ruppenthal , et al. 2013 Irisin and exercise training in humans ‐ results from a randomized controlled training trial. BMC Med. 11:235. 24191966 10.1186/1741-7015-11-235 PMC4228275

[phy213319-bib-0011] Hu, W. , R. Wang , J. Li , J. Zhang , and W. Wang . 2016 Association of irisin concentrations with the presence of diabetic nephropathy and retinopathy. Ann. Clin. Biochem. 53(Pt 1):67–74. 25814621 10.1177/0004563215582072

[phy213319-bib-0012] Huerta, A. E. , P. L. Prieto‐Hontoria , M. Fernandez‐Galilea , N. Sainz , M. Cuervo , J. A. Martinez , et al. 2015 Circulating irisin and glucose metabolism in overweight/obese women: effects of alpha‐lipoic acid and eicosapentaenoic acid. J. Physiol. Biochem. 71:547–558. 25820474 10.1007/s13105-015-0400-5

[phy213319-bib-0014] Huh, J. H. , S. V. Ahn , J. H. Choi , S. B. Koh , and C. H. Chung . 2016 High Serum Irisin Level as an Independent Predictor of Diabetes Mellitus: A Longitudinal Population‐Based Study. Medicine (Baltimore) 95:e3742. 27281072 10.1097/MD.0000000000003742 PMC4907650

[phy213319-bib-0013] Huh, J. Y. , G. Panagiotou , V. Mougios , M. Brinkoetter , M. T. Vamvini , B. E. Schneider , et al. 2012 FNDC5 and irisin in humans: I. Predictors of circulating concentrations in serum and plasma and II. mRNA expression and circulating concentrations in response to weight loss and exercise. Metabolism 61:1725–1738. 23018146 10.1016/j.metabol.2012.09.002 PMC3614417

[phy213319-bib-0015] Keuper, M. , M. Jastroch , C. X. Yi , P. Fischer‐Posovszky , M. Wabitsch , M. H. Tschop , et al. 2014 Spare mitochondrial respiratory capacity permits human adipocytes to maintain ATP homeostasis under hypoglycemic conditions. FASEB J. 28:761–770. 24200885 10.1096/fj.13-238725

[phy213319-bib-0016] Kim, M. K. , Y. N. Chae , M. H. Son , S. H. Kim , J. K. Kim , H. S. Moon , et al. 2008 PAR‐5359, a well‐balanced PPARalpha/gamma dual agonist, exhibits equivalent antidiabetic and hypolipidemic activities in vitro and in vivo. Eur. J. Pharmacol. 595:119–125. 18727927 10.1016/j.ejphar.2008.07.066

[phy213319-bib-0017] Kleiner, S. , R. J. Mepani , D. Laznik , L. Ye , M. J. Jurczak , F. R. Jornayvaz , et al. 2012 Development of insulin resistance in mice lacking PGC‐1alpha in adipose tissues. Proc. Natl. Acad. Sci. USA 109:9635–9640. 22645355 10.1073/pnas.1207287109 PMC3386123

[phy213319-bib-0018] Kurdiova, T. , M. Balaz , M. Vician , D. Maderova , M. Vlcek , L. Valkovic , et al. 2014 Effects of obesity, diabetes and exercise on Fndc5 gene expression and irisin release in human skeletal muscle and adipose tissue: in vivo and in vitro studies. J. Physiol. 592:1091–1107. 24297848 10.1113/jphysiol.2013.264655 PMC3948565

[phy213319-bib-0019] Lopez‐Legarrea, P. , R. de la Iglesia , A. B. Crujeiras , M. Pardo , F. F. Casanueva , M. A. Zulet , et al. 2014 Higher baseline irisin concentrations are associated with greater reductions in glycemia and insulinemia after weight loss in obese subjects. Nutr. Diabetes 4:e110. 24567125 10.1038/nutd.2014.7 PMC3940831

[phy213319-bib-0020] Moreno‐Navarrete, J. M. , F. Ortega , M. Serrano , E. Guerra , G. Pardo , F. Tinahones , et al. 2013 Irisin is expressed and produced by human muscle and adipose tissue in association with obesity and insulin resistance. J. Clin. Endocrinol. Metab. 98:E769–778. 23436919 10.1210/jc.2012-2749

[phy213319-bib-0021] National Research Council (US) Committee for the Update of the Guide for the Care and Use of Laboratory Animals (2011). Guide for the care and use of laboratory animals. National Academies Press Washington DC.

[phy213319-bib-0022] Norheim, F. , T. M. Langleite , M. Hjorth , T. Holen , A. Kielland , H. K. Stadheim , et al. 2014 The effects of acute and chronic exercise on PGC‐1alpha, irisin and browning of subcutaneous adipose tissue in humans. FEBS J. 281:739–749. 24237962 10.1111/febs.12619

[phy213319-bib-0023] Novelle, M. G. , C. Contreras , A. Romero‐Pico , M. Lopez , and C. Dieguez . 2013 Irisin, two years later. Int. J. Endocrinol. 2013:746281. 24298283 10.1155/2013/746281 PMC3835481

[phy213319-bib-0024] Nygaard, H. , G. Slettalokken , G. Vegge , I. Hollan , J. E. Whist , T. Strand , et al. 2015 Irisin in blood increases transiently after single sessions of intense endurance exercise and heavy strength training. PLoS ONE 10:e0121367. 25781950 10.1371/journal.pone.0121367 PMC4363689

[phy213319-bib-0025] Pardo, M. , A. B. Crujeiras , M. Amil , Z. Aguera , S. Jimenez‐Murcia , R. Banos , et al. 2014 Association of irisin with fat mass, resting energy expenditure, and daily activity in conditions of extreme body mass index. Int. J. Endocrinol. 2014:857270. 24864142 10.1155/2014/857270 PMC4016898

[phy213319-bib-0026] Park, K. H. , L. Zaichenko , M. Brinkoetter , B. Thakkar , A. Sahin‐Efe , K. E. Joung , et al. 2013 Circulating irisin in relation to insulin resistance and the metabolic syndrome. J. Clin. Endocrinol. Metab. 98:4899–4907. 24057291 10.1210/jc.2013-2373 PMC3849667

[phy213319-bib-0027] Roca‐Rivada, A. , C. Castelao , L. L. Senin , M. O. Landrove , J. Baltar , A. Belen Crujeiras , et al. 2013 FNDC5/irisin is not only a myokine but also an adipokine. PLoS ONE 8:e60563. 23593248 10.1371/journal.pone.0060563 PMC3623960

[phy213319-bib-0028] Schlogl, M. , P. Piaggi , S. B. Votruba , M. Walter , J. Krakoff , and M. S. Thearle . 2015 Increased 24‐hour ad libitum food intake is associated with lower plasma irisin concentrations the following morning in adult humans. Appetite 90:154–159. 25765248 10.1016/j.appet.2015.03.003 PMC4410111

[phy213319-bib-0029] Seo, D. Y. , H. B. Kwak , S. R. Lee , Y. S. Cho , I. S. Song , N. Kim , et al. 2014 Effects of aged garlic extract and endurance exercise on skeletal muscle FNDC‐5 and circulating irisin in high‐fat‐diet rat models. Nutr Res Pract 8:177–182. 24741402 10.4162/nrp.2014.8.2.177 PMC3988507

[phy213319-bib-0030] Sesti, G. , F. Andreozzi , T. V. Fiorentino , G. C. Mannino , A. Sciacqua , M. A. Marini , et al. 2014 High circulating irisin levels are associated with insulin resistance and vascular atherosclerosis in a cohort of nondiabetic adult subjects. Acta Diabetol. 51:705–713. 24619655 10.1007/s00592-014-0576-0

[phy213319-bib-0031] Sharma, N. , C. M. Castorena , and G. D. Cartee . 2012 Greater insulin sensitivity in calorie restricted rats occurs with unaltered circulating levels of several important myokines and cytokines. Nutr. Metab. (Lond) 9:90. 23067400 10.1186/1743-7075-9-90 PMC3541154

[phy213319-bib-0032] Shi, X. , M. Lin , C. Liu , F. Xiao , Y. Liu , P. Huang , et al. 2016 Elevated circulating irisin is associated with lower risk of insulin resistance: association and path analyses of obese Chinese adults. BMC Endocr. Disord. 16:44. 27473122 10.1186/s12902-016-0123-9 PMC4966722

[phy213319-bib-0033] Srinivasa, S. , C. Suresh , J. Mottla , S. R. Hamarneh , J. E. Irazoqui , W. Frontera , et al. 2016 FNDC5 relates to skeletal muscle IGF‐I and mitochondrial function and gene expression in obese men with reduced growth hormone. Growth Horm. IGF Res. 26:36–41. 26774404 10.1016/j.ghir.2015.12.008 PMC4716612

[phy213319-bib-0034] Stengel, A. , T. Hofmann , M. Goebel‐Stengel , U. Elbelt , P. Kobelt , and B. F. Klapp . 2013 Circulating levels of irisin in patients with anorexia nervosa and different stages of obesity–correlation with body mass index. Peptides 39:125–130. 23219488 10.1016/j.peptides.2012.11.014

[phy213319-bib-0035] Vamvini, M. T. , K. N. Aronis , G. Panagiotou , J. Y. Huh , J. P. Chamberland , M. T. Brinkoetter , et al. 2013 Irisin mRNA and circulating levels in relation to other myokines in healthy and morbidly obese humans. Eur. J. Endocrinol. 169:829–834. 24062354 10.1530/EJE-13-0276 PMC3857961

[phy213319-bib-0036] Wu, M. V. , G. Bikopoulos , S. Hung , and R. B. Ceddia . 2014 Thermogenic capacity is antagonistically regulated in classical brown and white subcutaneous fat depots by high fat diet and endurance training in rats: impact on whole‐body energy expenditure. J. Biol. Chem. 289:34129–34140. 25344623 10.1074/jbc.M114.591008 PMC4256346

[phy213319-bib-0037] Xin, C. , J. Liu , J. Zhang , D. Zhu , H. Wang , L. Xiong , et al. 2016 Irisin improves fatty acid oxidation and glucose utilization in type 2 diabetes by regulating the AMPK signaling pathway. Int. J. Obes. (Lond) 40:443–451. 26403433 10.1038/ijo.2015.199

[phy213319-bib-0038] Yang, Z. , X. Chen , Y. Chen , and Q. Zhao . 2015 Decreased irisin secretion contributes to muscle insulin resistance in high‐fat diet mice. Int. J. Clin. Exp. Pathol. 8:6490–6497. 26261526 PMC4525860

[phy213319-bib-0039] Zhang, S. , L. Yang , P. Chen , H. Jin , X. Xie , M. Yang , et al. 2016 Circulating Adipocyte Fatty Acid Binding Protein (FABP4) Levels Are Associated with Irisin in the Middle‐Aged General Chinese Population. PLoS ONE 11:e0146605. 26752184 10.1371/journal.pone.0146605 PMC4709139

